# A composite index for predicting improvement of mitral regurgitation in patients with multivalvular heart disease after transcatheter aortic valve replacement

**DOI:** 10.3389/fcvm.2025.1679115

**Published:** 2025-11-03

**Authors:** Zhi-Cheng Xiao, Gui-Bin Zheng, Tian-Xiang Geng, Zhong-Fei Lu, Zhen Wang, Lei Zheng, Meng-Meng Ren, Fa-Xin Ren

**Affiliations:** ^1^ Department of Cardiology, Yantai Yuhuangding Hospital Affiliated to Medical College of Qingdao University, Yantai, Shandong, China; ^2^ Department of Thyroid Surgery, Yantai Yuhuangding Hospital Affiliated to Medical College of Qingdao University, Yantai, Shandong, China; ^3^ Department of Orthopedics, Yantai Yuhuangding Hospital Affiliated to Medical College of Qingdao University, Yantai, Shandong, China; ^4^ State Key Laboratory of Cardiovascular Disease, Department of Radiology, Fuwai Hospital, National Center for Cardiovascular Diseases, Chinese Academy of Medical Sciences and Peking Union Medical College, Beijing, China; ^5^ Department of Cardiac Surgery, Yantai Yuhuangding Hospital Affiliated to Medical College of Qingdao University, Yantai, Shandong, China

**Keywords:** aortic stenosis, transcatheter aortic valve replacement, mitral regurgitation, atrial fibrillation, eccentric regurgitation

## Abstract

**Background:**

Transcatheter aortic valve replacement (TAVR) has become a standard treatment for severe aortic stenosis (AS). Concomitant mitral regurgitation (MR) is observed in approximately 19%–29% of these patients. Although MR frequently improves after TAVR, persistent MR is associated with worse clinical outcomes, highlighting the need for reliable predictors of MR persistence.

**Methods:**

We conducted a single-center, retrospective study that included 53 patients with severe AS and moderate-to-severe MR who underwent TAVR between 2017 and 2024. The primary outcome was MR improvement, defined as a reduction of at least one grade at the 1-year follow-up. To identify independent predictors, we performed multivariable logistic regression analysis.

**Results:**

At 1-year follow-up, MR improvement was observed in 67.9% of patients. Multivariable analysis identified persistent atrial fibrillation [odds ratio (OR): 0.099, 95% CI: 0.017–0.575; *P* = 0.01] and eccentric MR (OR: 0.066, 95% CI: 0.012–0.370; *P* = 0.002) as independent negative predictors of improvement. Conversely, greater interventricular septal thickness (OR: 1.825, 95% CI: 1.075–3.099; *P* = 0.026) was a positive predictor. A composite predictive index integrating these three variables demonstrated excellent discriminative ability, with an area under the receiver operating characteristic curve of 0.909.

**Conclusion:**

This study identifies persistent atrial fibrillation, eccentric MR, and interventricular septal thickness as key independent predictors of MR improvement after TAVR. The derived composite index, with its outstanding predictive performance, provides a novel tool to aid in patient selection and prognostic assessment.

## Introduction

Transcatheter aortic valve replacement (TAVR) has become the mainstay of treatment for symptomatic severe aortic stenosis (AS), particularly for patients with high surgical risk or elderly patients (1–4). Concomitant moderate to severe mitral regurgitation (MR) occurs in approximately 19%–29% of TAVR recipients, with 44%–58% demonstrating MR improvement after the procedure (5–7). Notably, MR improvement after TAVR is associated with significantly better survival outcomes compared with persistent severe MR (8, 9).

For patients unlikely to achieve MR improvement, combined valvular surgery may represent a more optimal therapeutic strategy; however, accurately identifying this subgroup remains clinically challenging. Prior studies have proposed several predictors of MR improvement, including a mean transaortic gradient of ≥40 mmHg, functional MR etiology, and the absence of pulmonary hypertension or atrial fibrillation (AF) (6). Conversely, persistent MR has been linked to primary mitral regurgitation, a larger D-shaped annular circumference (10), mitral apparatus calcification (11), the female sex, a lower body mass index, and elevated right ventricular systolic pressure (12).

Despite these findings, no validated predictive model currently exists to guide personalized management for patients with concomitant aortic and mitral valve disease. To address this gap, this study seeks to identify the predictors of MR improvement following TAVR and develop a composite index using retrospective data from our institution.

## Methods

### Study design and patient selection

This single-center, retrospective cohort study enrolled consecutive patients with symptomatic severe AS and concomitant moderate to severe MR who underwent TAVR at our hospital between October 2017 and May 2024. Key exclusion criteria included (1) perioperative death and (2) loss to follow-up or missing post-TAVR echocardiographic data. Treatment plans were determined by a multidisciplinary heart team after obtaining informed patient consent. Patients with moderate to severe mitral stenosis or a flail mitral leaflet were typically referred for combined surgery, unless deemed ineligible. The final cohort comprised 53 patients. Ethical approval was obtained from the Ethics Committee of Yantai Yuhuangding Hospital (No. 2025–597), with individual consent waived in accordance with the Declaration of Helsinki. All patients received self-expanding valves, including the Venus A-Valve (Venus Medtech, Hangzhou, Zhejiang, China), VitaFlow Valve (MicroPort, Shanghai, China), and TaurusOne Valve (Peijia Medical, Suzhou, Jiangsu, China). Transthoracic echocardiography (TTE) was performed at baseline, after TAVR (within several days), and at 1 year following TAVR. MR improvement was defined as a reduction of ≥1 grade in severity. Acute kidney injury (AKI) and bleeding were defined as a ≥50% increase in serum creatinine and a hemoglobin decrease of ≥30 g/L, respectively.

### Echocardiography and MSCT assessment

TTE was performed using the Philips EPIQ 7C system. Severe AS was defined as a mean gradient of ≥40 mmHg and a peak velocity of ≥4 m/s (or an aortic valve area ≤0.8 cm^2^ in low-flow, low-gradient cases). MR severity was graded mainly based on regurgitant jet width (mild: <3 mm; moderate: 3–6.9 mm; and severe: ≥7 mm). Primary MR was defined as a structural abnormality of the mitral valve leaflets or subvalvular apparatus. To ensure unbiased assessment, all echocardiograms were independently reviewed by two experienced echocardiographers who were blinded to all clinical data, patient outcomes, and each other's assessments. In cases of discrepancy (e.g., a difference of ≥1 grade), a third senior reader (adjudicator) provided a final adjudicated grade.

Cardiac evaluation was performed using multislice computed tomography (MSCT) scanners [SOMATOM Force (Siemens Healthineers, Erlangen, Germany) and Optima CT660 (GE Healthcare, Waukesha, WI, USA)]. Iohexol contrast (60–80 mL) was administered intravenously at a controlled rate. Image acquisition was performed with a slice thickness of 0.5 mm for cardiac structures with ECG synchronization and 1 mm for aortic assessment. The annular dimensions of the aortic valve were analyzed using 3Mensio software. Mitral annular parameters—including D-shaped annular circumference, trigone-to-trigone distance, anteroposterior distance, commissural distance, and calcification length—were measured using Siemens Healthineers software, as reported previously (10). The left atrial and ventricular volumes were quantified at 35%–40% and 70%–75% of the RR interval, respectively. The spherical index was calculated as the ratio of the left ventricular long-axis to short-axis diameter (10).

### Statistical analysis

Data were analyzed using IBM SPSS Statistics version 26 and R version 4.4.3. Normally distributed continuous variables were expressed as mean ± standard deviation (SD) and compared using the Student's independent t-test. Categorical data were presented as frequencies and percentages [*n* (%)] and analyzed using Pearson's chi-square or Fisher's exact test. Non-normally distributed variables were reported as median (interquartile range, IQR) and compared using the Mann–Whitney *U* test. Variables showing significant differences between the two groups of patients were further assessed via univariable analysis. Variables with *p* <0.05 in the univariable analysis were entered into the multivariable logistic regression model. Odds ratios (ORs) with 95% confidence intervals (CIs) were calculated to evaluate the associations between independent variables and endpoint events. A composite index was derived from significant predictors, and its discriminative ability was evaluated using receiver operating characteristic (ROC) curve and decision curve analyses. A two-sided *p*-value <0.05 was considered statistically significant.

## Results

### Baseline characteristics

The cohort included 53 consecutive patients (mean age 74.8 ± 6.9 years, 43.4% male), of whom 36 patients (67.9%) showed MR improvement at 1 year. The risk score of the Society of Thoracic Surgeons (STS) was 4.4 ± 3.1%. Coronary artery disease was present in 47.2% of the patients, with 26.4% undergoing concurrent percutaneous coronary intervention. Approximately 67.9% of the patients were grouped as New York Heart Association (NYHA) class III. Persistent AF was a significantly more frequent occurrence in the non-improvement group (47.1% vs. 11.1%, *P* = 0.015) (Table 1).

**Table 1:** Baseline characteristics.

Characteristic	Total (*n* = 53)	No improvement (*n* = 17)	Improvement (*n* = 36)	*P*-value
Age, year	74.8 ± 6.9	76.8 ± 7.5	73.8 ± 6.5	0.15
Male, *n* (%)	23 (43.4)	8 (47.1)	15 (41.7)	0.71
Primary mitral regurgitation, *n* (%)	4 (7.6)	1 (5.9)	3 (8.3)	0.75
STS score, %	4.4 ± 3.1	5.3 ± 3.6	4.0 ± 2.8	0.15
Hypertension, *n* (%)	29 (54.7)	10 (58.8)	19 (52.8)	0.68
Diabetes mellitus, *n* (%)	11 (20.8)	5 (29.4)	6 (16.7)	0.29
Chronic obstructive pulmonary disease, *n* (%)	5 (9.4)	2 (11.8)	3 (8.3)	0.69
Coronary artery disease, *n* (%)	25 (47.2)	9 (52.9)	16 (44.4)	0.63
Coronary intervention, *n* (%)	14 (26.4)	6 (35.3)	8 (22.2)	0.31
Atrial fibrillation, *n* (%)	16 (30.2)	9 (52.9)	7 (19.4)	0.026
Paroxysmal atrial fibrillation, *n* (%)	4 (7.6)	1 (5.9)	3 (8.3)	1
Persistent atrial fibrillation, *n* (%)	12 (22.6)	8 (47.1)	4 (11.1)	0.015
Peripheral artery disease, *n* (%)	6 (11.3)	4 (23.5)	2 (5.6)	0.08
Cerebrovascular disease, *n* (%)	6 (11.3)	3 (17.7)	3 (8.3)	0.37
NYHA functional class, *n* (%)				0.57
II	1 (1.9)	0 (0)	1 (2.8)	
III	36 (67.9)	13 (76.5)	23 (63.9)	
IV	16 (30.2)	4 (23.5)	12 (33.3)	
Creatinine, μmol/L	71.4 ± 19.6	70.2 ± 13.5	72.0 ± 22.1	0.76
Cancer, *n* (%)	4 (7.6)	2 (11.8)	2 (5.6)	0.59

STS, The Society of Thoracic Surgeons; NYHA: New York Heart Association.

A majority of the patients (79.3%) had moderate MR. Concomitant moderate aortic regurgitation and severe aortic regurgitation were observed in 52.83% and 18.87% of the patients, respectively. Four patients (7.5%) had mild mitral stenosis, and one had mitral valve prolapse (without a flail leaflet). Eccentric MR was significantly more prevalent in the non-improvement group (70.6% vs. 22.2%, *P* < 0.05). No significant differences were observed in left ventricular diameters or stroke volume between the improvement and the non-improvement groups. However, the improvement group demonstrated greater interventricular septal thickness (13.2 ± 1.9 vs. 12.0 ± 1.9 mm, *P* = 0.033) and posterior wall thickness (11.4 ± 1.8 vs. 10.5 ± 1.0 mm, *P* = 0.046). Bicuspid aortic valves were also more common in the improvement group (25.0% vs. 5.9%, *P* = 0.047). No significant differences were found in mitral annular dimensions, calcification length, or atrial/ventricular volumes (Table 2).

**Table 2:** Cardiac ultrasound and MSCT data.

Characteristic	Total (*n* = 53)	No improvement (*n* = 17)	Improvement (*n* = 36)	*P*-value
Aortic regurgitation grade, *n* (%)				0.63
0	4 (7.6)	1 (5.9)	3 (8.3)	
1	11 (20.8)	2 (11.8)	9 (25.0)	
2	28 (52.8)	11 (64.7)	17 (47.2)	
3	10 (18.9)	3 (17.7)	7 (19.4)	
Mitral regurgitation grade, *n* (%)				0.7
2	42 (79.3)	14 (82.4)	28 (77.8)	
3	11 (20.8)	3 (17.7)	8 (22.2)	
Mitral stenosis, *n* (%)	4 (7.6)	1 (5.9)	3 (8.3)	1
Eccentric MR, *n* (%)	20 (37.7)	12 (70.6)	8 (22.2)	<0.001
Left ventricular diameter, mm	52.6 ± 6.4	54.2 ± 6.5	51.9 ± 6.3	0.21
Left ventricular end-diastolic diameter, mm	58.3 ± 6.9	58.1 ± 5.8	58.4 ± 7.4	0.89
Left ventricular end-systolic diameter, mm	41.7 ± 8.9	41.5 ± 6.0	41.8 ± 10.0	0.89
Left ventricular end-systolic volume, mL	168.0 ± 50.3	168.5 ± 42.5	167.8 ± 54.2	0.96
Stroke volume, mL	89.5 ± 33.9	90.1 ± 20.8	89.2 ± 38.9	0.92
Left atrial diameter, mm	47.3 ± 6.1	49.8 ± 7.00	46.2 ± 5.4	0.047
Interventricular septum thickness, mm	12.8 ± 1.9	12.0 ± 1.9	13.2 ± 1.9	0.033
Left ventricular posterior wall thickness, mm	11.1 ± 1.6	10.5 ± 1.0	11.4 ± 1.8	0.046
Left ventricular ejection fraction, %	50.7 ± 11.6	53.5 ± 8.0	49.3 ± 12.8	0.22
Mean aortic gradient, mmHg	56.1 ± 18.6	52.9 ± 19.3	57.6 ± 18.4	0.40
Mean aortic velocity, m/s	4.8 ± 0.7	4.6 ± 0.8	4.8 ± 0.7	0.38
Tricuspid regurgitation grade, *n* (%)				0.42
0	2 (3.8)	1 (5.9)	1 (2.8)	
1	25 (47.2)	6 (35.3)	19 (52.8)	
2	21 (39.6)	7 (41.2)	14 (38.9)	
3	5 (9.4)	3 (17.7)	2 (5.6)	
Pulmonary artery pressure, mmHg	44.2 ± 16.2	44.2 ± 16.4	44.3 ± 16.3	0.99
Mitral valve prolapse, *n* (%)	1 (1.9)	1 (5.9)	0	0.32
Bicuspid aortic valve, *n* (%)	10 (18.9)	1 (5.9)	9 (25.0)	0.047
Trigone-to-trigone distance, mm	2.6 ± 0.3	2.6 ± 0.3	2.6 ± 0.4	0.67
Intercommissural distance, mm	3.6 ± 0.4	3.6 ± 0.4	3.6 ± 0.4	0.91
Anteroposterior distance, mm	2.8 ± 0.4	2.8 ± 0.4	2.8 ± 0.4	0.70
Annular circumference, mm	10.9 ± 1.0	10.9 ± 1.0	10.8 ± 1.0	0.83
Annular area, mm^2^	8.8 ± 1.6	9.0 ± 1.8	8.7 ± 1.5	0.60
Annular circumference/trigone-to-trigone distance, mm	4.2 ± 0.5	4.1 ± 0.4	4.2 ± 0.50	0.60
Posterior leaflet calcification, *n* (%)	2 (3.8)	1 (5.9)	1 (2.8)	0.59
Mitral valve annulus calcification, *n* (%)	12 (22.6)	2 (11.8)	10 (27.8)	0.16
Intercommissural calcification, *n* (%)	5 (9.4)	2 (11.8)	3 (8.3)	0.70
Calcification length, mm (Q1, Q3)	0.00 (0.00, 1.1)	0.00 (0.00, 0.00)	0.00 (0.00, 1.1)	0.73
Left atrial volume, mL	162.5 ± 48.7	180.5 ± 65.5	154.3 ± 37.0	0.15
Left ventricular volume, mL	151.7 ± 60.6	150.6 ± 66.0	152.1 ± 59.0	0.93
Left atrial volume/annular circumference	14.9 ± 4.2	16.5 ± 5.8	14.2 ± 3.2	0.07
Left ventricular volume/annular circumference	13.9 ± 5.2	13.7 ± 5.4	14.0 ± 5.2	0.84
Sphere index	1.6 ± 0.3	1.5 ± 0.3	1.6 ± 0.2	0.24

MSCT, multislice computed tomography; MR, mitral regurgitation.

The distribution of calcification length was non-normal; data are presented as median (Q1–Q3).

### Operative procedures and outcomes

Perioperative complications were moderate paravalvular leaks and new-onset complete atrioventricular block. The leaks occurred in seven patients (13.21%); permanent pacemaker implantation was required in seven other patients because of new-onset complete atrioventricular block. Other complications included stroke (3.77%), acute kidney injury (3.77%), and bleeding (9.43%). Aortic peak velocity following TAVR was higher in the improvement group (2.35 ± 0.61 vs. 2.02 ± 0.40 m/s, *P* = 0.045) than in the non-improvement group. One-year readmission and mortality rates were 13.21% (primarily due to heart failure or stroke) and 3.77% (attributed to sudden death and pneumonia), respectively (Table 3). Among the 36 patients who showed a reduction in mitral regurgitation, the extent of improvement followed a graded distribution: One patient improved by three grades, five patients by two grades, and the remaining thirty by one grade (data not available).

**Table 3:** Perioperative data and clinical outcomes at 1 year.

Characteristic	Total (*n* = 53)	No improvement (*n* = 17)	Improvement (*n* = 36)	*P*-value
Paravalvular leakage grade, *n* (%)				0.52
0	36 (67.9)	13 (76.5)	23 (63.9)	
1	10 (18.9%)	3 (17.7%)	7 (19.4%)	
2	7 (13.2)	1 (5.9)	6 (16.7)	
Postoperative aortic velocity, m/s	2.2 ± 0.6	2.0 ± 0.4	2.4 ± 0.6	0.045
Left bundle branch block	8 (15.1)	3 (17.7)	5 (13.9)	0.72
Atrioventricular conduction block	7 (13.2)	2 (11.8)	5 (13.9)	1
Permanent pacemaker implantation	9 (17.0)	4 (23.5)	5 (13.9)	0.63
Size of prosthetic valves	26.1 ± 2.5	26.6 ± 2.7	25.8 ± 2.4	
Type of prosthetic valves				0.7
1	32 (60.4)	9 (52.9)	23 (63.9)	
2	19 (35.9)	7 (41.2)	12 (33.3)	
3	2 (3.8)	1 (5.9)	1 (2.8)	
Valve-in-valve, *n* (%)	1 (1.9)	0	1 (2.8)	1
Mechanical support device, *n* (%)	3 (5.7)	0	3 (8.3)	0.54
Perioperative stroke, *n* (%)	2 (3.8)	0	2 (5.6)	1
Acute renal failure, *n* (%)	2 (3.8)	0	2 (5.6)	1
Hemorrhage, *n* (%)	5 (9.4)	0	5 (13.9)	0.16
Heart failure, *n* (%)	2 (3.8)	1 (5.9)	1 (2.8)	0.54
Readmission, *n* (%)	7 (13.2)	2 (11.8)	5 (13.9)	1
Death, *n* (%)	2 (3.8)	0	2 (5.6)	1
Cerebral ischemic stroke, *n* (%)	4 (7.7)	1 (6.3)	3 (8.3)	1

### Predictors of mitral regurgitation improvement

**Figure 1:**
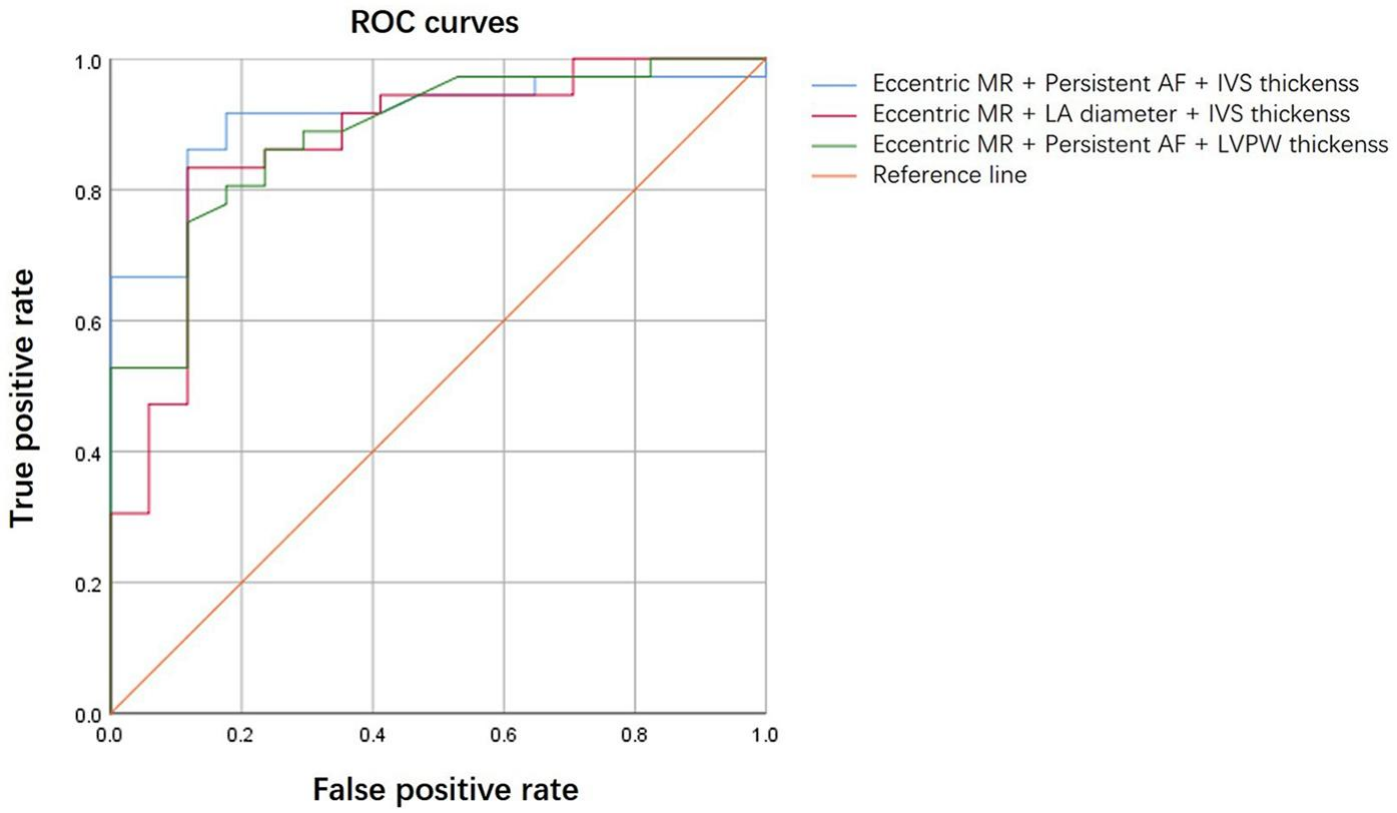
ROC curves comparing composite indices.

**Figure 2:**
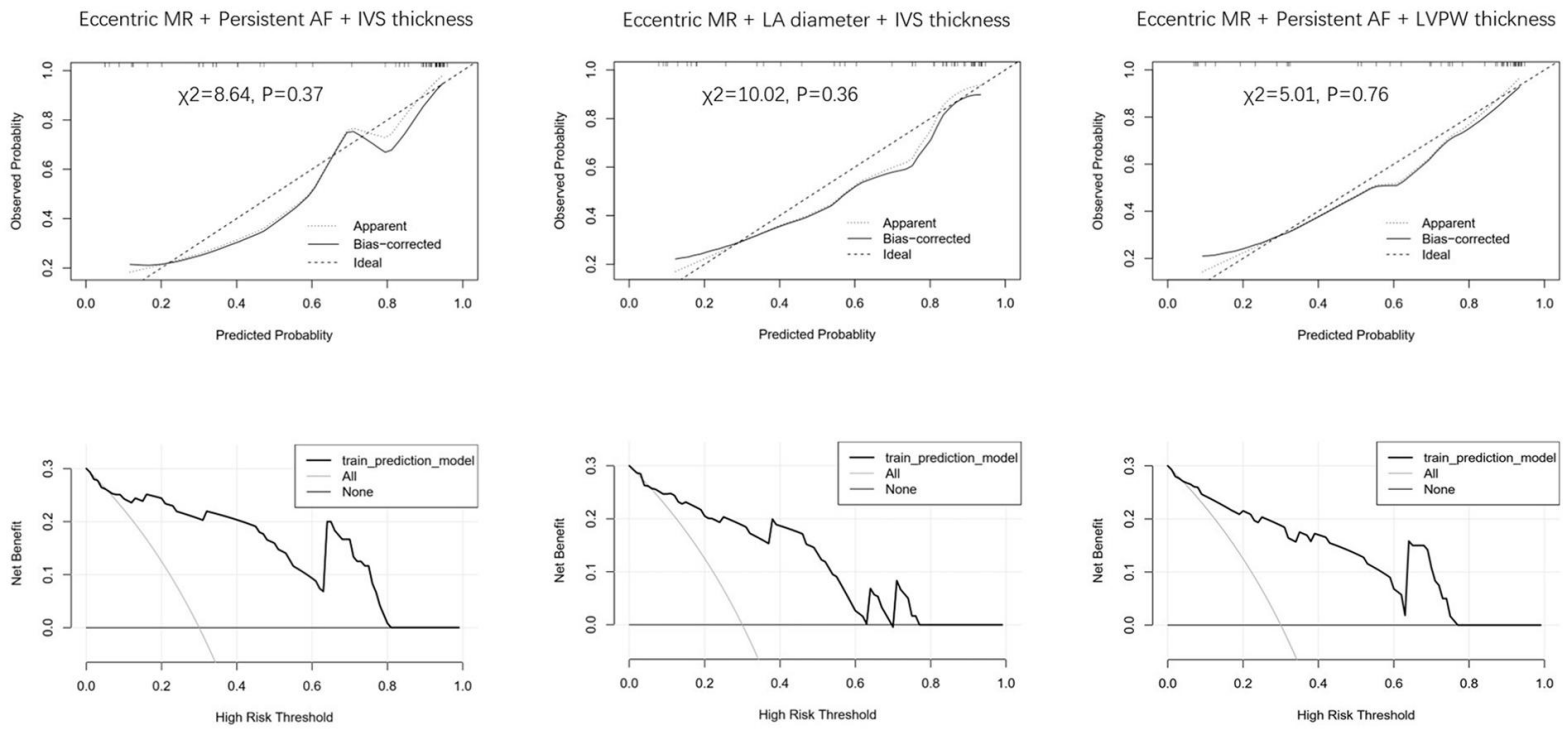
Calibration and decision curve analysis for different indices.

The logistic regression analysis included persistent AF, left atrial diameter, interventricular wall (IVS) thickness, left ventricular posterior wall (LVPW) thickness, eccentric MR, and bicuspid aortic valve. Persistent AF [OR: 0.14 (95% CI: 0.03–0.58), *P* = 0.006] and eccentric MR [OR: 0.12 (95% CI: 0.03–0.44), *P* = 0.001] were independent negative predictors of MR improvement. In contrast, IVS thickness emerged as a positive predictor of MR improvement [OR: 1.45 (95% CI: 1.01–2.08), *P* = 0.044] (Table 4). The combination of eccentric MR, persistent AF, and IVS thickness constituted a powerful predictor [area under the curve (AUC) = 0.909 (95% CI: 0.83–0.99), *P* < 0.001] (Figure 1). The optimal cut point for this index was 0.507, yielding the highest overall accuracy, as reflected by the greatest Youden Index (*J* = 0.74). At this threshold, the index demonstrated excellent clinical utility: The positive predictive value (PPV) was 91.7% (95% CI: 62.0%–99.8%) and the negative predictive value (NPV) was 82.4% (95% CI: 59.0%–94.0%) (Table 5). Because atrial fibrillation was significantly correlated with the left atrial diameter, and interventricular septal thickness was highly correlated with left ventricular posterior wall thickness, we developed composite indices by combining these parameters with eccentric regurgitation, which demonstrated comparable predictive values without statistical significance (Figure 1 and Table 5, Supplementary Table 1). Model validation results demonstrated favorable predictive performance. The Hosmer–Lemeshow test indicated a robust goodness of fit (*P* > 0.05 for all three composite indices). The calibration curves showed that both the apparent and the bias-corrected curves closely followed the ideal diagonal line, confirming the accuracy of the predicted probabilities. The decision curve analysis further revealed that the use of our model for clinical decision-making yielded a higher net benefit across a wide range of threshold probabilities (0.1–0.8) compared with the “treat all” or “treat none” strategies (Figure 2).

**Table 4:** Logistic regression analysis of indicators.

Indicators	Univariable analysis	*P*-value	Multivariable analysis	*P*-value
	Odds ratio		Odds ratio	
Persistent atrial fibrillation	0.14 (0.03–0.58)	0.006	0.099 (0.017–0.575)	0.01
Left atrial diameter	0.91 (0.82–1.00)	0.056		
Interventricular septum thickness	1.45 (1.01–2.08)	0.044	1.825 (1.075–3.099)	0.026
Posterior wall thickness of the left ventricle	1.59 (0.99–2.56)	0.054		
Eccentric mitral regurgitation	0.12 (0.03–0.44)	0.001	0.066 (0.012–0.370)	0.002
Bicuspid aortic valve	5.33 (0.62–46.09)	0.128		

Persistent atrial fibrillation, interventricular septum thickness, and eccentric mitral regurgitation were included in the multivariable analysis.

**Table 5:** ROC curve analysis of the composite indices.

Composite index	AUC (95% CI)	Asymptotic sig.	Cut point	Sensitivity	Specificity	Youden index	PPV (95% CI)	NPV (95% CI)
Eccentric MR + persistent AF + IVS thickness	0.909 (0.83–0.99)	<0.001	0.507	0.917	0.824	0.740	91.7% (78.6%–97.5%)	82.4% (59.0%–94.0%)
Eccentric MR + LA diameter + IVS thickness	0.871 (0.76–0.98)	<0.001	0.698	0.833	0.882	0.716	93.8% (80.8%–98.4%)	71.4% (50.0%–86.2%)
Eccentric MR + persistent AF + LVPW thickness	0.884 (0.79–0.98)	<0.001	0.711	0.750	0.882	0.632	93.1% (78.5%–98.2%)	62.5% (42.5%–79.2%)

MR, mitral regurgitation; AF, atrial fibrillation; IVS, interventricular septum; LA, left atrial; LVPW, left ventricular posterior wall; AUC, area under the curve; CI, confidence interval; PPV, positive predictive value; NPV, negative predictive value.

## Discussion

It is well recognized that the etiologies of mitral regurgitation are not mutually exclusive. A mixed etiology, characterized by the coexistence of organic pathology and functional mechanisms, is observed in a substantial proportion of patients (13). Notably, MR grade improvement has been reported in 59% of patients with organic MR (14). In consonance with this, in our cohort, 7.55% of patients presented with rheumatic mitral valve changes and mild mitral stenosis. In these mixed-etiology MR cases, although organic structural abnormalities persist after TAVR, the functional components often improve, resulting in overall MR reduction. Our findings revealed that 67.9% of patients with moderate to severe MR experienced postprocedural MR reduction at 1 year—a rate consistent with that of previous reports (15).

Atrial fibrillation drives left atrial remodeling, which can lead to mitral annular dilation. Although the mitral leaflets may enlarge adaptively to compensate initially, excessive dilation impairs leaflet coaptation, resulting in functional mitral regurgitation (16). Prior studies have identified both new-onset and preexisting atrial fibrillation as negative predictors of MR improvement (11, 17). Consistent with these reports, our analysis confirmed a significant association between AF and reduced MR improvement; however, subgroup stratification revealed that only persistent AF remained a statistically significant predictor, whereas paroxysmal AF did not. We hypothesize that persistent AF induces a more pronounced remodeling, making the resultant MR less reversible after TAVR.

Another finding was that eccentric mitral regurgitation was negatively correlated with MR improvement. Eccentric regurgitation is known to constitute the vast majority of cases involving primary MR. Recent studies have indicated that eccentric regurgitation is also relatively frequent in ventricular functional mitral regurgitation (VFMR), whereas its prevalence is lower in atrial functional mitral regurgitation (AFMR). Notably, AFMR demonstrates a higher likelihood of improvement after TAVR compared with VFMR (7). In VFMR, an eccentric jet typically arises from the relative overshoot of one leaflet, resulting in a genuine eccentrically directed jet (18). In AFMR, however, an apparent eccentric jet may result from left atrial dilatation and posterior mitral annular displacement onto the crest of the left ventricular inlet. This anatomical shift can cause a centrally originating jet to adhere to the adjacent atrial wall because of the Coanda effect, giving the impression of an eccentric jet rather than a truly asymmetric one (19). Thus, the presence of an eccentric jet may often reflect organic pathology or result from irreversible ventricular or atrial remodeling, under which circumstances MR is less likely to resolve. Alternatively, it may represent a pseudo-eccentric jet induced by phenomena such as the Coanda effect. Consistent with this, our results demonstrated a negative correlation between eccentric regurgitation and post-TAVR MR improvement.

Ventricular reverse remodeling is observed after TAVR and is likely associated with MR improvement (20). Moreover, ventricular reverse remodeling has been corroborated by a study of surgical aortic valve replacement (SAVR), which showed that more than 50% of patients exhibited a significant decrease (>15%) in indexed left ventricular mass. Patients with pronounced reverse remodeling were also likely to have had more severe baseline parameters of aortic stenosis (21). Consistent with this, Fabián Islas's staging system categorizes patients with AS based on left ventricular global longitudinal strain, the presence of MR, and right ventricular–arterial coupling. Their work demonstrates that patients at later stages exhibit greater left ventricular mass and a higher likelihood of post-TAVR cardiac function improvement, suggesting an association between advanced remodeling and the potential for recovery (22). In our study, interventricular septal thickness, as an indicator of ventricular mass, was correlated with MR improvement after TAVR. Greater IVS thickness may be indicative of more advanced disease stages and represent greater ventricular remodeling potential. However, limited data precluded formal cardiac staging in our cohort. In addition, postprocedural increased aortic velocity may represent a secondary consequence of mitigated mitral regurgitation. Because it is not a preoperative parameter, it was not included in the univariable analysis.

Several predictors, such as secondary MR, LV end-diastolic diameter, and reduced left ventricular ejection fraction (LVEF), have been reported to be associated with MR improvement after TAVR. Alternatively, other factors, including primary MR, left atrial area, mitral annular diameter ≥35 mm, and pulmonary hypertension, have been negatively associated with MR improvement (15). In addition, a D-shaped annular circumference has been linked to reduced MR improvement (10). Perioperative and postoperative factors can also influence MR improvement. For instance, residual AR has been correlated with worsening MR (17, 23), and excessively deep implantation of the valve may impair the motion of the anterior mitral leaflet, potentially exacerbating MR (24). Despite the numerous influencing factors, no single factor can reliably predict changes in postprocedural MR. Given the diverse mechanisms underlying mitral regurgitation, composite indicators may offer better predictive accuracy. For instance, Li et al. achieved exceptional prediction (AUC: 0.91) using multiple algorithms, with shapley additive explanations (SHAP) analysis identifying moderate to severe MR, leaflet thickening, ejection fraction, pulmonary hypertension, and left atrial size as the top contributors (25). Similarly, our composite index—which incorporates persistent AF, eccentric MR, and interventricular septal thickness—demonstrated not only excellent discriminative ability (AUC: 0.909) but also robust internal validation. More importantly, the use of our model could help optimize clinical decision-making by more effectively identifying those patients most likely to benefit from TAVR in terms of MR improvement, thereby maximizing net benefit compared with universal strategies. These findings strengthen the rationale for using persistent AF, eccentric MR, and IVS thickness as key predictors in such integrated models.

### Study limitations and conclusion

The key limitations of this study include its modest cohort size (*n* = 53), which raises concerns regarding potential overfitting, and the lack of advanced imaging metrics such as CT-derived extracellular volume or longitudinal strain (26). Furthermore, the model was validated only on an internal dataset and therefore lacks external validation, which limits the generalizability of the findings. Despite these constraints, our study provides clinicians with a practical, evidence-based tool for pre-TAVR assessment of complex AS-MR patients.

In conclusion, the composite index integrating persistent atrial fibrillation, eccentric mitral regurgitation patterns, and interventricular septal thickness demonstrates robust predictive value for post-TAVR MR improvement, facilitating optimized therapeutic decision-making. Although these preliminary findings are clinically promising, they require validation through large-scale prospective multicenter studies.

## Data Availability

The datasets presented in this study are available from the corresponding author upon reasonable request and are subject to approval by the institutional ethics committee.

## Supplementary material

Supplementary Table S1 Comparison of ROC curve predictive performance.
