# Supplementary material for: A composite index for predicting improvement of mitral regurgitation in patients with multivalvular heart disease after transcatheter aortic valve replacement
Source: Front Cardiovasc Med. 2025 Nov 3;12:1679115. doi: 10.3389/fcvm.2025.1679115 (PMC12620972; doi:10.3389/fcvm.2025.1679115)
Supplement: Supplementary Table S1 — Comparison of ROC curve predictive performance. [file Table1.docx]

| **Supplemental Table 1. Comparison of ROC curve predictive performance** | | | |
| --- | --- | --- | --- |
| Test Pair | Asymptotic Sig. | AUC Difference | Std. Error Difference |
| A vs. B | 0.437 | 0.038 | 0.307 |
| A vs. C | 0.300 | 0.025 | 0.292 |
| B vs. C | 0.811 | -0.013 | 0.316 |
| A: Eccentric MR + Persistent AF + IVS thickness; B: Eccentric MR + LA diameter + IVS thickness; C: Eccentric MR + Persistent AF + LVPW thickness | | | |
